# Phosphodiesterase 8A to discriminate in blood samples depressed patients and suicide attempters from healthy controls based on A-to-I RNA editing modifications

**DOI:** 10.1038/s41398-021-01377-9

**Published:** 2021-04-30

**Authors:** Nicolas Salvetat, Fabrice Chimienti, Christopher Cayzac, Benjamin Dubuc, Francisco Checa-Robles, Pierrick Dupre, Sandie Mereuze, Vipul Patel, Catherine Genty, Jean-Philippe Lang, Jean-François Pujol, Philippe Courtet, Dinah Weissmann

**Affiliations:** 1 ALCEDIAG/Sys2Diag, CNRS UMR 9005, Parc Euromédecine, Cap Delta, 1682 Rue de la Valsière, Montpellier, 34184 France; 2 Department of Emergency Psychiatry and Acute Care, University Hospital/INSERM U1061, 191 Av. du Doyen Gaston Giraud, Montpellier, 34295 France

**Keywords:** Molecular neuroscience, Diagnostic markers

## Abstract

Mental health issues, including major depressive disorder, which can lead to suicidal behavior, are considered by the World Health Organization as a major threat to global health. Alterations in neurotransmitter signaling, e.g., serotonin and glutamate, or inflammatory response have been linked to both MDD and suicide. Phosphodiesterase 8A (PDE8A) gene expression is significantly decreased in the temporal cortex of major depressive disorder (MDD) patients. PDE8A specifically hydrolyzes adenosine 3′,5′-cyclic monophosphate (cAMP), which is a key second messenger involved in inflammation, cognition, and chronic antidepressant treatment. Moreover, alterations of RNA editing in PDE8A mRNA has been described in the brain of depressed suicide decedents. Here, we investigated PDE8A A-to-I RNA editing-related modifications in whole blood of depressed patients and suicide attempters compared to age-matched and sex-matched healthy controls. We report significant alterations of RNA editing of PDE8A in the blood of depressed patients and suicide attempters with major depression, for which the suicide attempt took place during the last month before sample collection. The reported RNA editing modifications in whole blood were similar to the changes observed in the brain of suicide decedents. Furthermore, analysis and combinations of different edited isoforms allowed us to discriminate between suicide attempters and control groups. Altogether, our results identify PDE8A as an immune response-related marker whose RNA editing modifications translate from brain to blood, suggesting that monitoring RNA editing in PDE8A in blood samples could help to evaluate depressive state and suicide risk.

## Introduction

Suicidal behavior and suicide are a major public health issue worldwide; suicide is one of the primary causes of death, with an estimated 1 million deaths every year^1^. About 90% of suicide completers suffered from mental disorders; half of them met the current criteria for major depressive disorder^2^. Single genes variants might not explain the full risk of developing suicidal behavior^3^, genome-wide association studies for MDD failed to identify causative variants^4^. However, changes at the molecular level are detectable in brains of depressive patients and suicide completers. Modifications in serotonin receptor and transporter expression, both of which resulting in altered serotonergic signaling, were the first to be described^5^. Additional molecular changes in MDD include other neurotransmitters pathways, e.g., glutamate and gamma-aminobutyric acid^6^. Alterations of inflammatory response have also been linked to both MDD and suicide (for review see ref. ^7^). A close interaction between immune system activation and changes in brain circuits related to mood and behavior has been described^8^. Deterioration of the normal structure and function of microglia, possibly caused by inflammatory signals, can lead to depression^9^. It is now admitted that depressed individuals display dysregulation of immune system (for review see ref. ^10^). Very recently, an association between pro-inflammatory cytokines and brain activation has been identified in patients with MDD and vulnerability to suicide^11^. A significant increase in depressive symptoms was observed in subjects injected with Lipopolysaccharide from *Salmonella abortus*, suggesting a direct link between immune response and mood alteration^12^. Occurrence of severe depression with suicidal ideation in patients undergoing interferon (IFN) treatment is well documented (for review see ref. ^13^), suggesting strong connections between inflammation and depression. Inflammation can modify dopaminergic, serotoninergic, and glutamatergic systems (for review see ref. ^14^).On the opposite, type I interferon signaling was recently shown to be altered in blood cells of patients suffering from severe recurrent depression^15^.

Transcriptomic and epi-transcriptomic studies have recently identified peripheral markers of suicide risk (for review see ref. ^16^). Significant differences in the methylation patterns associated with astrocytic alterations have been described in depression and suicide^17^. In the central nervous system (CNS), RNA editing is an essential cellular mechanism for diversifying the transcriptome. Introducing codon changes in mRNAs encoding key proteins for nervous system activity, including glutamate and serotonin receptors, has been shown to modulate their activity^18^. Major changes in RNA editing patterns have been correlated with neuronal disease pathogenesis^19^. Adenosine Deaminase Acting on RNA (ADARs), the enzymes responsible for A-to-I editing of RNAs, have been implicated in immune regulation^20^. Interestingly, the p150 isoform of ADAR1 (ADAR1a) is induced by a variety of inflammatory mediators, including TNF-alpha and IFN-alpha^21^. Upregulated expression of ADAR1 was also observed in the dorsolateral prefrontal cortex of major depressive suicide victims^22^. We have previously shown that region-specific alterations of A-to-I RNA editing of 5HT2CR occur in different areas of the prefrontal cortex in suicide decedents^23^. More recently, region-specific RNA editing alterations of an immune response marker, phosphodiesterase 8A (PDE8A), were described in the cortex of suicides with major depression^24^. Cyclic nucleotide phosphodiesterases are a family of enzymes which selectively hydrolyze cAMP, cGMP or both, and thus affect G-protein-coupled signal transduction (for review see ref. ^25^). The pre-mRNA of PDE8A has previously been shown to be post transcriptionally edited by ADARs^26^ in T cells from systemic lupus erythematosus (SLE) patients and in T cells activated with type I interferon^27^. There is a high prevalence of depression in SLE patients^28^.

The aim of the study is to evaluate in blood RNA editing modifications of PDE8A, already described in brain, in depressed patients. We have investigated alterations of PDE8A RNA editing in the blood of controls, depressed patients and suicide attempters with major depression and identified specific combinations to discriminate between these groups.

## Materials and methods

### Subjects and clinical assessment

This monocentric study was conducted in the CHRU of Montpellier according to the principles of the Helsinki Declaration of 1975 and its successive updates, and was approved by the French local Ethical Committee (CPP No. A01978-41). The samplings were performed under the expertise of Prof. Philippe Courtet, in charge of the Psychiatry collection of the Centre de Resources Biologiques at Montpellier Hospital.

Participants (MDD = DEP + SA), were recruited from September 2016 to January 2019 among the outpatients of the Department of Emergency Psychiatry and Post-Acute Care (CHRU of Montpellier). Age-matched, race-matched, and sex-matched control subjects were recruited from a list of volunteers from the Clinical Investigation Center (CHRU of Montpellier). All participants, aged between 18 and 65 years, understood and signed a written informed consent before entering the study.

The study included three groups: i) healthy controls (Ctrl, *n* = 99), which includes individuals with no history of any major DSM-IV axis I disorder and of suicidal behavior/act; ii) depressed patients (DEP; *n* = 101):depressed patients without any records of suicidal act lifetime and iii/ Suicide attempters (SA; *n* = 84): depressed patients with a history of at least one suicide attempt (defined as any act carried out with some intent to die) during the last month before sample collection. All patients met the MDD criteria in Diagnostic and Statistical Manual of Mental disorders IV (DSM-IV) using the Mini-International Neuropsychiatric Interview. During the standardized interview, psychiatrists managed the French version of the Montgomery-Åsberg Depression Rating Scale (MADRS)^29^ and the 30-item Inventory of Depressive Symptomatology, Clinician Rated (IDS-C30)^30^ to score the depression. Depression severity levels, i.e., low, moderate, and severe were defined by 7 ≤ MADRS ≤ 19 and/or 12 ≤ IDSC-30 ≤ 23, 20 ≤ MADRS ≤ 34 and/or 24≤IDSC-30 ≤ 36, MADRS ≥ 35 and/or IDSC-30 ≥ 37, respectively. The severity of the most severe suicide attempt was evaluated with the Risk Rescue Rating Scale (RRRS)^31^, which measures the medical danger of the attempt (risk factors) and the probability of being rescued (rescue factors), and the 19-item Suicide Intent Scale (SIS), which evaluate the intent to die.

### RNA extraction and qualification from whole blood

Samples were retrieved in PAXgene™ blood RNA tubes, which are optimized for stabilization of RNA, and distributed randomly in the different sets of extractions. Total RNAs were isolated using the MagNA Pure 96 Cellular RNA Large Volume Kit (LifeScience), according to the manufacturer’s protocol. During sample preparation and RNA extraction, standard precautions were taken to avoid RNA degradation by RNAses. Total RNA concentrations and quality levels were determined with Qubit Fluorometer (Invitrogen) and LabChip (Perkin-Elmer, HT RNA Reagent Kit) instruments, respectively. Only samples with RIN number >7 were taken into consideration for further analysis.

### Reverse transcription and quantitative real-time PCR

Reverse transcription was carried out on 12.5 ng/µL of RNA per sample using the Takara kit (PrimeScript RT, Takara). The resulting cDNA was combined with TaqMan universal PCR Master Mix (Applied Biosystems) and with the following specific gene probes: ADAR1-1 (ADAR1a-p150) (Hs01020780), ADAR1-5 (ADAR1-b-p110) (Hs01017596), ADAR2 (Hs00210562), PDE8A (Hs00400174), GAPDH (Hs02758991), HPRT1 (Hs02800695), TBP (HS00427620) and PGK1 (Hs99999906) (Life Technologies) in 20 µL final volume. Quantitative PCRs were performed in 384-well plates on LightCycler 480 Real Time PCR instrument (Roche). The analysis was performed using a second derivative absolute quantification, normalized by the geometric mean of four housekeeping genes (HPRT1, GAPDH, TBP, and PGK1).

### Next generation sequencing (NGS) library preparation and sequencing

For NGS library preparation, we chose a targeted approach to selectively sequence the region of interest within intron 9 of the PDE8A gene (Suppl Fig. 1A). Validated PCR primers were used to amplify the region of interest by PCR (Suppl Fig. 2). NGS library preparation has been detailed elsewhere^32^. Experiments were performed five times independently for each sample.

### Bioinformatics analysis of sequencing data

The sequencing data were downloaded from the NextSeq500 sequencer (Illumina) and demultiplexed as fastq file. The procedure for sequencing quality evaluation, trimming, and alignment has been described previously^32^. Calling of edited position was run using an in-house script, which counts the number of different nucleotides in each genomic location (“base count”). For each position, the script computes the percentage of reads that have a “G” [Number of “G” reads / (Number of “G” reads + Number of “A” reads) * 100]. The genomic location “A” reference with percentage in “G” reads > 0.1 are automatically detected by the script and are considered as “A-to-I edition site” (Suppl Fig. 1B). The last stage was to compute the percentage of all possible isoforms of PDE8A transcripts. By definition the relative proportion of RNA editing at a given editing “site” represents the sum of editing modifications measured at this unique genomic coordinate. An mRNA isoform is a unique molecule that may or may not contain multiple editing modifications on the same transcript. For example, the PDE8A mRNA isoform BC contains a modification on both site B and site C within the same transcript. A relative proportion of at least 0.1% was set as the threshold in order to be included in the analysis.

### Statistical analysis of data

All statistics and figures were computed with the “R/Bioconductor” statistical open source software^33^. RNA editing values are usually presented as means ± standard error of the mean (SEM). A differential analysis was carried out with the non-parametric Wilcoxon rank sum test and the Welch’s *t*-test. With the multiple testing methodologies, it is important to adjust the *p*-value of each editing isoforms to control the false discovery rate (FDR). The Benjamini and Hochberg (BH) procedure^34^ was applied on all statistical tests with the “multtest package” and an adjusted *p*-value below 0.05 was considered as statistically significant. Relative proportion of editing levels was normally distributed and consequently no normalization was applied.

A random forest (RF) approach was applied to assess the RNA editing isoform combinations of PDE8A mRNA transcript. The concept of aggregating the results of many decision trees has resulted in a stable algorithm, robust to noisy data^35^. Random forest require the use of a training set used to build the model and a test set to validate it. We have shared our data set: 2/3 of the dataset (*n* = 125) are used for the learning phase and 1/3 are used for the test phase (*n* = 50). This sharing has been randomized and respect the initial proportion of the various statutes in each sample. To estimate RF parameters, we used the 10-fold cross-validation method. For these approaches, we used the “randomForest package 4.6-14” of the R software version 3.5.3 with mtry = 2, ntree = 1000 as parameters for the model. Results of best RF model selected are represent by ROC curve and diagnostic performance on test phase.

## Results

### Subjects

A paired case–control design was used to control demographic and assay variance. Potential confounding factors such as sex, age, educational level, or BMI were not statistically different between groups (Table 1). The inflammation marker CRP was not statistically different between groups, though there was a trend to higher CRP in suicide attempters. We observed a marked association in substance abuse disorders in depressed patients (Table 1). Two different clinical evaluations were considered to confirm the presence or absence of psychiatric disorders, namely MADRS and IDS-C30. We observed a significant association (*r*^2^ = 0.90, *p* < 0.0005) between the two evaluations (Suppl Fig. 3), suggesting a correct assignment of patients and a relative homogeneity in the scoring of depression. Depressed patients (DEP: depressed patients without any history of suicide and SA: suicide attempters) had significantly higher scores in both MADRS and IDSC-30 than controls (Table 1). We chose to discard the small group of low severity depression patients (Table 1) from the analysis to work on a group of patients with a well-characterized depressive state (MADRS > 20 or IDSC30 > 24). All patients were treated, and treatments were classified into five categories: antidepressant, antipsychotics, anxiolytics, hypnotics/sedatives and antiepileptics. Treatments categories did no significantly differ between DEP and SA groups (Table 1). Principal Component Analysis for the four categories of treatments did not show a significant clustering of patients (Suppl Fig. 4), suggesting that the possible impact of treatments on the modifications of RNA editing would be to be similar for both DEP and SA groups.

**Table 1:** Demographic characteristics of participants and psychiatric diagnosis of patients.

	Total sample	Controls	DEP	SA
*Demographics*				
	*n*	*n*	*n*	*n*
Number	284	99	101	84
Age (min–max)	18–67	18–64	18–67	18–60
Age (mean ± SD)	39.3 (±13.5)	41 (±14.4)	40.1 (±12.9)	36.3 (±12.9)
*p* value (vs. Ctrl)			0.63	0.022
*p* value (vs. DEP)				0.051
*Gender*				
Male (*n*(%))	102 (35.9%)	40 (40.4%)	39 (38.6%)	23 (27.4%)
Female (*n*(%))	182 (64.1%)	59 (59.6%)	62 (61.4%)	61 (72.6%)
CRP (mean ± SEM)	3 (±0.5)	2.4 (±0.5)	2.3 (±0.5)	4.7 (±1.3)
*p* value (vs. Ctrl)			0.89	0.097
*p* value (vs. DEP)				0.086
BMI (mean ± SD)	23.89 (±4.79)	24.4 (±4.1)	23.47 (±4.5)	23.7 (±5.87)
Years of education (mean ± SD)	14.01 (±2.43)	14.3 (±2.3)	14.15 (±2.27)	13.12 (±2.77)
*Substances addiction*				
Tobacco smoking (*n* (%))	111 (39.1%)	16 (16.2%)	46 (45.5%)	49 (58.3%)
*p* value (vs.DEP)				0.76
Alcohol use disorder (*n* (%))	31 (10.9%)	0 (0%)	15 (14.9%)	16 (19%)
*p* value (vs. DEP)				1
Other substances use disorder (*n* (%))	19 (6.7%)	0 (0%)	9 (8.9%)	10 (11.9%)
*p* value (vs. DEP)				1
*Psychotropic treatments*				
Anxiolytics (*n* (%))	–	NA	55 (54.5%)	51 (60.7%)
Hypnotics and sedatives (*n* (%))	–	NA	14 (13.9%)	16 (19%)
Antidepressants (*n* (%))	–	NA	60 (59.4%)	53 (63.1%)
Antipsychotics (*n* (%))	–	NA	40 (39.6%)	26 (31%)
Antiepileptics (*n* (%))	–	NA	22 (21.8%)	15 (17.9%)
Mean treatment uptake (mean ± SEM)	–	NA	2.1 (±0.1)	2.2 (±0.2)
*p* value (vs. DEP)				0.65
*Clinical characteristics*				
Average MADRS score (mean ± SD)	0.65 (±1.25)	26.03 (±10.14)	28.99 (±9.55)	23.94 (±10.99)
Average IDSC30 score (mean ± SD)	2.01 (±2.46)	32.2 (±11.25)	34.87 (±10.02)	32.73 (±12.18)
*p* value (vs. Ctrl)			<0.0001	<0.0001
*Degree of severity*				
No depression (*n* (%))	–	99 (100%)	0	–
Low severity (*n* (%))	–	–	19 (18.81%)	8 (9.52%)
Moderate severity (*n* (%))	–	–	41 (40.59%)	36 (42.86%)
High severity (*n* (%))	–	–	31 (30.69%)	30 (35.71%)
Very high severity (*n* (%))	–	–	10 (9.90%)	10 (11.90%)

Data are the mean ± SEM. *p*-values of main characteristics are displayed with the Student’s *t*-test. *p*-value of psychotropic treatments are displayed with the chi-squared-test.

*CTRL* healthy controls, *DEP* depressive patients, *SA* suicide attempters, *BMI* body mass index.

### Characterization of PDE8A pre-mRNA intron 9 editing sites

**Fig. 1:**
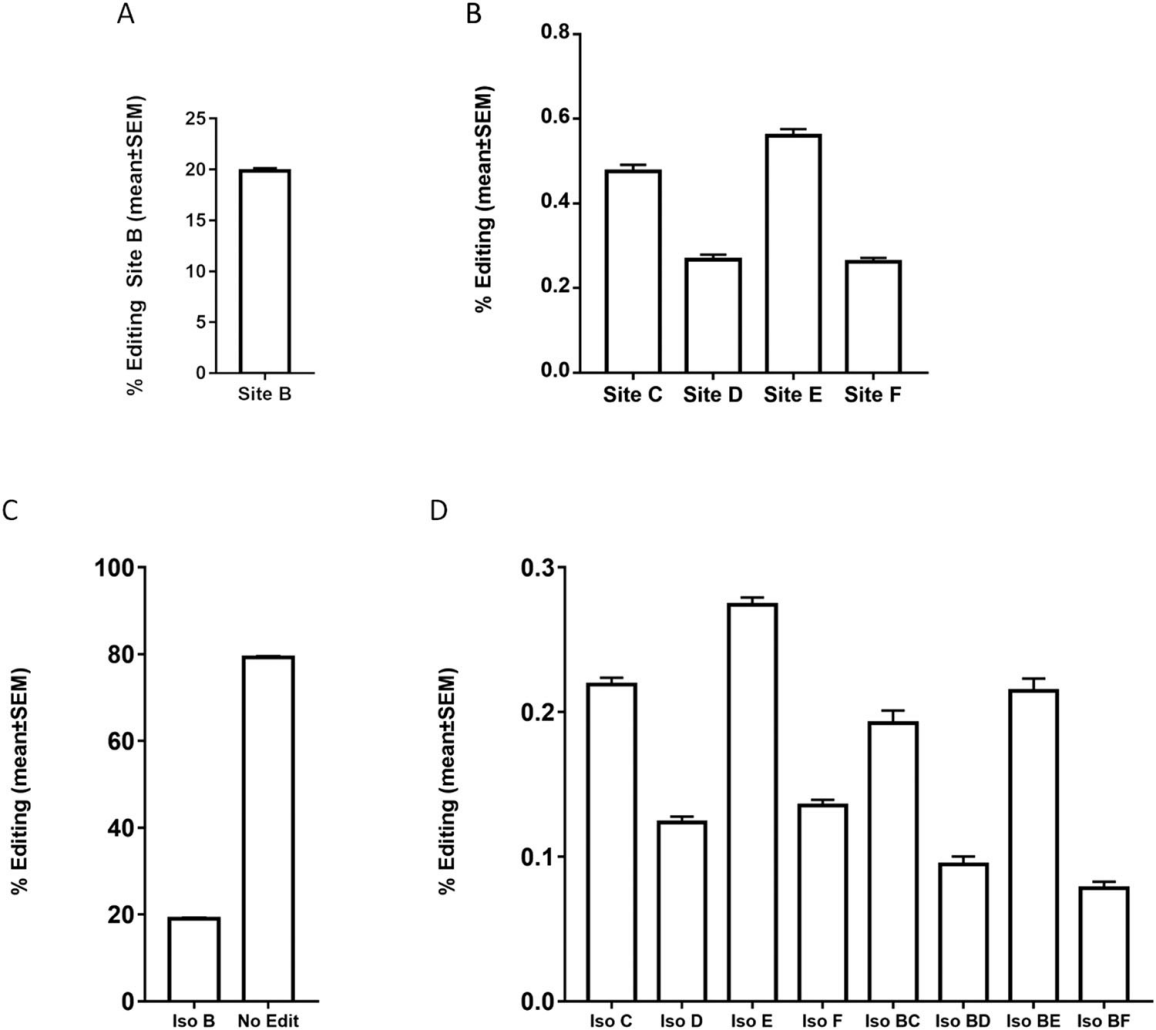
Distribution of PDE8A mRNA sites and isoforms measured in whole blood on samples of the control group. Histograms represent relative editing proportion (%) of the detected PDE8A editing site (**A**, **B**) or isoform (**C**, **D**) (mean ± s.e.m.; *n* = 99). Only isoforms representing more than 0.1% of relative proportion were included in the analysis.

In the 225 bp sequence studied here in whole blood, we observed the presence of five sites whose editing rates were above the 0.1% threshold (Fig. 1A). Sites A, G, H, I J, K, L, M and N (Suppl Fig. 2) were only observed with a very low editing rate, e.g., <0.05% and were therefore excluded from further analysis. We observed a high rate of editing at site B, i.e., approx 20% editing (Fig. 1A), with a similar editing ratio to previously described in the brain. The other four sites considered were consistently edited in all samples, with editing ratios between 0.2–0.6% (Fig. 1B).

Overall, we observed a comparable profile with isoforms of PDE8A mRNA (Fig. 1C, D). Isoform B was the most prominent isoform, all other isoforms being edited below 0.5%. Similar to editing sites, isoforms C, D, E and F were present at low levels. Interestingly, editing at site B was present in all the isoforms; we did not detect isoforms of more than one site without site B being edited.

### MDD-induced specific changes in PDE8A mRNA editing

**Fig. 2:**
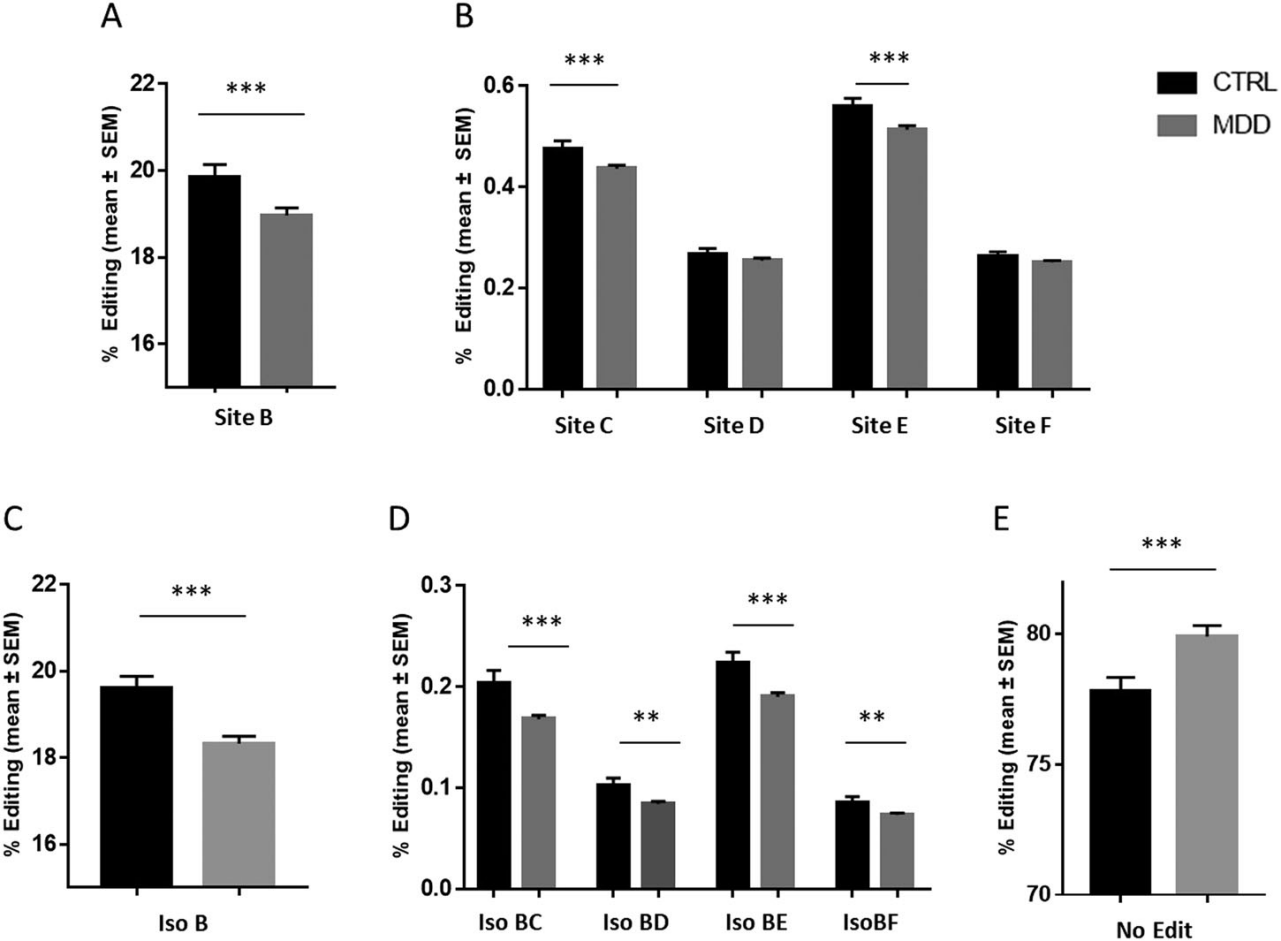
Relative proportion of mRNA PDE8A editing sites and isoforms measured in controls and depressed (MDD) patients. Histograms represent relative editing proportion (%) of the detected PDE8A editing site (**A**, **B**) or isoform (**C**–**E**) in healthy controls (mean ± s.e.m.; *n* = 99) or in the MDD group (*n* = 158). Only isoforms representing more than 0.1% of relative proportion were included in the analysis. Black: Controls, dark grey: MDD. The symbol * indicates a *p*-value ≤ 0.05, **p-value ≤ 0.01, and ***p-value ≤ 0.001.

We first run an analysis of the relative mRNA-editing profile of PDE8A in two distinct groups, i.e., healthy controls and depressed patients (MDD, DEP + SA). We identified a significant decrease of editing rates at sites B, C, and E between healthy controls and depressed patients (MDD, DEP + SA, Fig. 2A, B). Sites D and F were also decreased, though the decline was not statistically significant. When we compared isoforms rather than editing sites alone we found that isoforms B, BC, BD, BE, and BF were significantly decreased in patients vs. healthy controls (Fig. 2C, D). Strikingly, the decrease in either site or isoform B was mirrored by a significant increase in the non-edited isoform, i.e., mRNA isoform with no editing at all (Fig. 2E). The editing levels for all sites edited above the 0.1% threshold were strongly correlated between each other, in both controls and MDD groups (Suppl Fig. 5),suggesting that the modifications in editing levels occur in concert within the whole editing island of PDE8A mRNA.

### Specific changes in PDE8A mRNA editing measured in controls, depressed patients, and suicide attempters

**Fig. 3:**
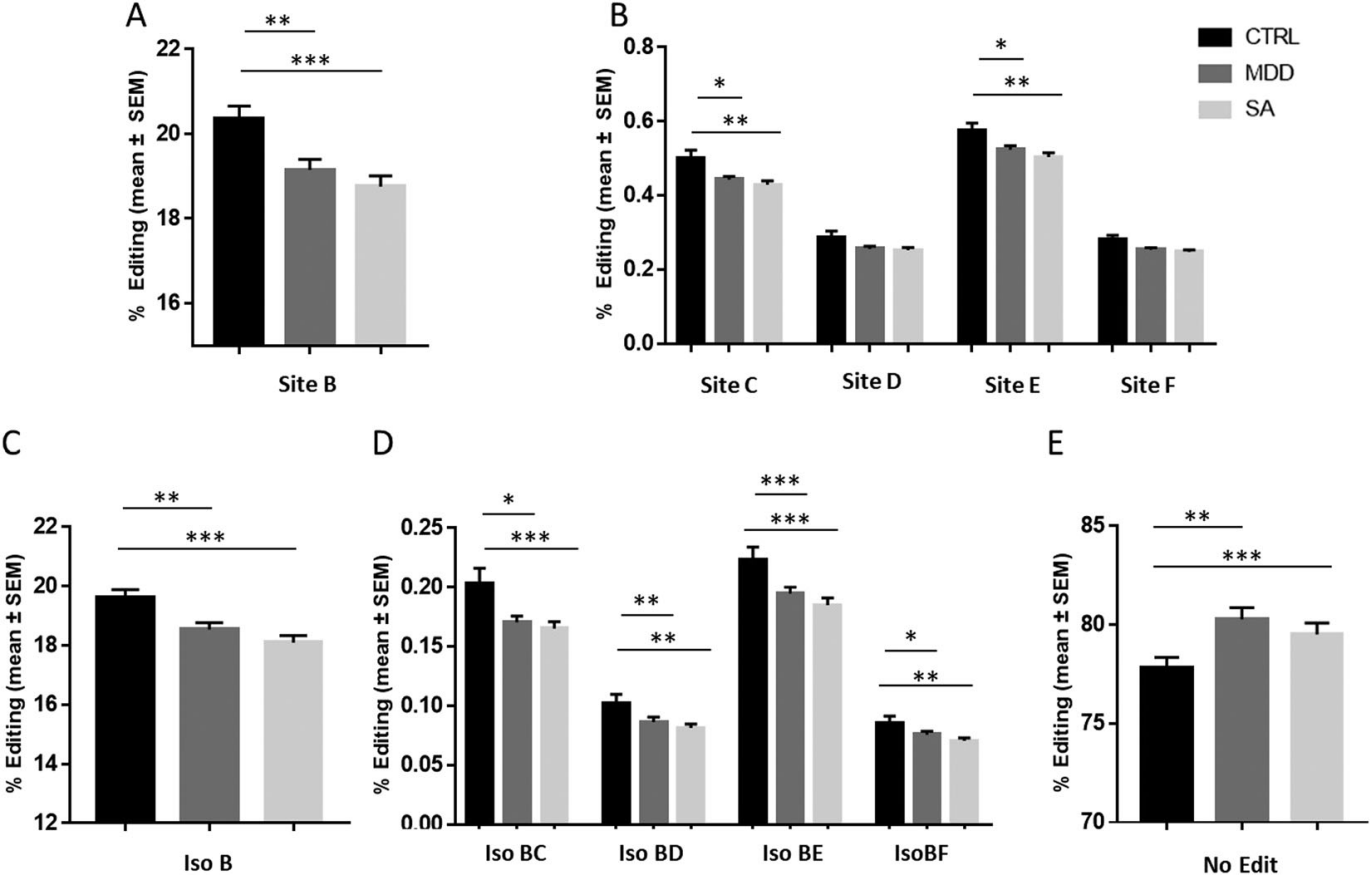
Relative proportion of mRNA PDE8A editing sites and isoforms measured in controls, depressed patients and suicide attempters. Histograms represent relative editing proportion (%) of the detected PDE8A editing site (**A**, **B**) or isoform (**C**–**E**) in healthy controls (mean ± s.e.m.; *n* = 99), DEP group (*n* = 82), or SA (*n* = 76). Only isoforms representing more than 0.1% of relative proportion were included in the analysis. Black: Controls, Dark grey: DEP; Light grey: SA. The symbol asterisk *p-value ≤ 0.05, **p-value ≤ 0.01, ***p-value ≤ 0.001.

The same analysis was performed on the sub-populations of depressed patients, namely DEP and SA, and compared to the control group. Parallel to the previous analysis, PDE8A RNA editing site B was found to be differentially edited between healthy controls and both DEP and SA, with a significant decrease observed in depressed patients (Fig. 3A). A trend of decrease between DEP and SA was observed, which did not reach statistical significance. PDE8A RNA editing levels on site C and E were also significantly decreased between healthy controls, DEP and SA (Fig. 3B). There was a trend of decrease between controls and patients for site D and F, though this decrease did not reach statistical significance.

Editing levels in PDE8A isoforms B, BC, BD, BE, and BF were significantly decreased in the two sub groups DEP and SA vs. healthy controls (Fig. 3C, D). Here again, the trend of decrease between DEP and SA was not significant. Interestingly, we observed a significant increase for the non-edited isoform in patients, either DEP or SA, (Fig. 3E), which mirrored the changes in isoform B in the opposite direction. As PDE8A RNA editing site B accounts for the vast majority of all edited position, it is likely that the change in editing observed is due to a change in ADAR recruitment rather that a global modification of editing positions.

Overall, these results indicate a significant decrease in PDE8A RNA editing rate between depressed patients and healthy controls. Interestingly, we noted a trend to a further decrease in suicide attempters vs. depressed patients with no history of suicide, though the diminution was not significant.

### Comparison of PDE8A RNA editing modifications in blood and brain

**Fig. 4:**
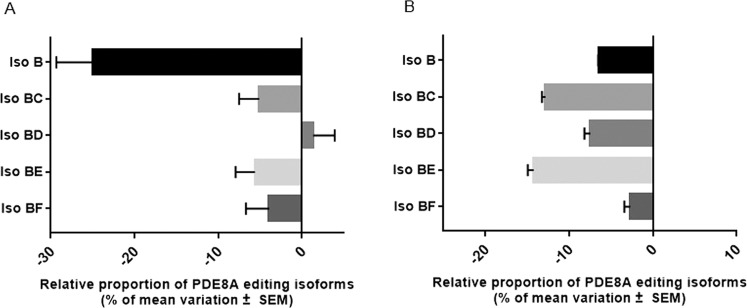
Comparison of modifications in PDE8A common isoforms between brain of suicide decedents and blood of suicide attempters. **A** Mean variation (mean ± S.E.M.) of PDE8A editing isoforms in the brain (Dorsolateral prefrontal cortex, BA24) between suicide decedents suffering major depressive disorder (*n* = 8) vs. healthy controls (*n* = 8). **B** Mean variation (mean ± S.E.M.) of PDE8A editing isoforms in the blood of suicide attempters (*n* = 76) vs. healthy controls (*n* = 99).

A previous study identified significant variations in PDE8A in the brain of depressed suicide decedents^24^. We thus aimed at comparing the changes in the relative proportions of PDE8A isoforms in blood and brain. As we detected more edited isoforms above the 0.1% threshold in the brain, we only matched the common isoforms identified in both tissues. We compared the changes observed in percentage of mean variations of different isoforms in the brain area BA24 of depressed suicide decedents with the variations identified in the blood of suicide attempters. Strikingly, the directions on variations were similar for all isoforms identified in the blood (Fig. 4). Isoform B decreased in blood of depressed patients, as in brain of suicide decedents, though to a lesser extent in blood than in the brain area BA24 (−22% in the brain vs. −6.4% in blood). Contrariwise, the combined isoforms BC, BE, and BF decreased approximately twice as much in the blood of depressed patients than in brain of suicide decedents. Isoform BD was significantly decreased in blood, while it remains quite stable in brain.

Overall, we observed that the changes in editing rates of PDE8A isoforms measured in depression and/or suicide were very similar in blood and brain.

### PDE8A and ADAR deaminases expression levels

Because A-to-I editing can have significant effect on target gene expression, and vice-versa, as to get more information of expression levels of the RNA editing enzymes, we quantified expression levels of PDE8A, ADAR1a, ADAR1B, and ADAR2 in our patient population.

PDE8A mRNA expression was stable between all groups, and we did not detect significant changes in expression (data not shown), suggesting that differential editing in intron 9 has no effect on mRNA levels. When we measured expression levels of the ADAR deaminases, we could not identified any significant modification in expression of ADAR1a (ADAR1-1, p150), ADAR1b (ADAR1-5, p110) or ADAR2 when we compared depressed patients or suicide attempters vs healthy controls (data not shown). As MDD has no effect on enzyme expression in the blood, these data suggest that the changes observed in editing rates are more likely due to modifications in ADAR activity or recruitment during depressive episodes.

### Predictive algorithm to detect patients with increased risk to have mood alterations

**Fig. 5:**
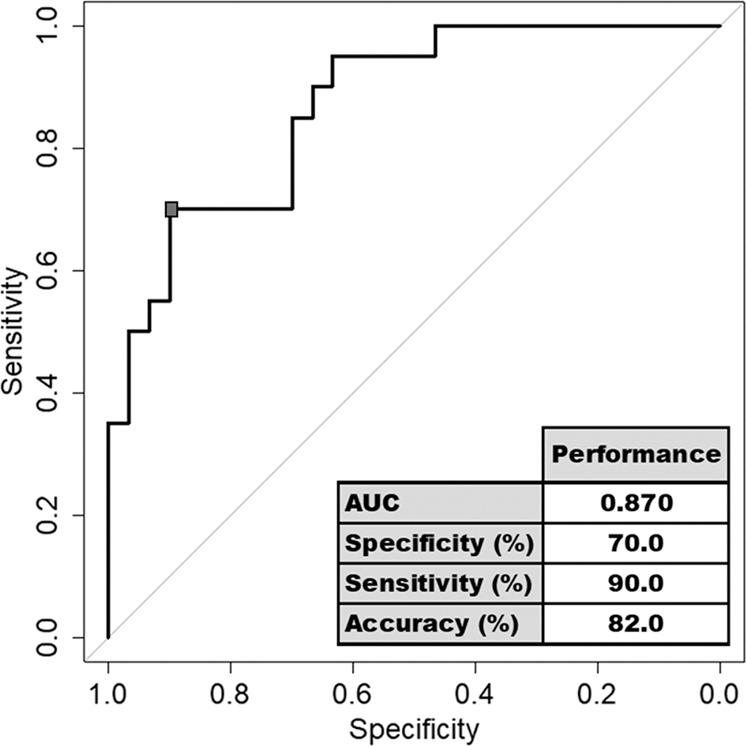
Diagnostic performances of a combination of PDE8A isoforms which separates suicide attempters from controls. ROC curve of a combination of PDE8A sites and isoforms, which separate the population of suicide attempters (*n* = 76) from healthy controls (*n* = 99). Diagnostic performances of the algorithm are indicated on the graph.

To evaluate their potentials as diagnostic test to predict suicide risk, all significant RNA Editing isoforms and sites were combined using random Forest (RF) machine learning approach. The diagnostic performance of RF model to separate suicide attempters from controls displayed an AUC ROC = 0.870 [0.774; 0.966] (Fig. 5), a specificity of 90% and a sensitivity of 70%. Overall, the identified RNA editing modifications in PDE8A mRNA isolated from blood samples allow accurate and robust detection of depressed patients with history of suicide attempts from controls.

## Discussion

In this study, we used targeted Next-Generation Sequencing to study the PDE8A RNA editing profile in whole blood from controls, depressed patients with no history of suicide attempt and suicide attempters. We observed a marked association in substance abuse disorders in depressed patients, which is particularly common and well documented in depression^36^. All patients included in the study were treated with different medications. Treatments were classified into five categories, the distribution of the five classes of treatments among patients being not statistically different between DEP and SA groups. Treatments might however represent a possible bias in the measurement of RNA editing. Some antipsychotic or antidepressant drugs, e.g., aripiprazole, sertindole, or nortriptyline tested in a cellular mode (SHSH-5Y cells) have been shown to modify the RNA editing profile of the 5HT2CR and to induce an editing profile similar to the one obtained after IFN-alpha treatment^37^. Indeed, IFN-alpha is known to induce ADAR1-1(p150) expression and increase editing of 5HT2CR in SHSH-5Y cells^37^. A recent work shown that PDE8A RNA editing is altered in both HCV patients following interferon alpha treatment and in IFN-alpha-treated SHSY-5Y neuroblastoma cells^32^. In the 225 bp studied in intron 9 of PDE8A, we identified significant alterations of PDE8A RNA editing in both depressed patients and suicide attempters. Editing levels were significantly decreased in all depressed patients, regardless of previous suicide attempt. Of note, suicide attempters had a trend to even lower editing levels when compared to DEP without history of suicide, though the decrease did not reach statistical significance. This decrease could be attributed to both pathology and pharmacology. As the five classes of medications did not cluster in a subgroup of patients, it is likely that the effect of treatments on RNA editing is uniformly distributed between patient groups.

We have previously described alterations in both 5HT2CR and PDE8A RNA editing in the brain of unmedicated suicide decedents^23,24^. Moreover, PDE8A RNA editing modifications were shown to be related to depression status in patients with chronic hepatitis C virus (HCV) infection undergoing antiviral combination therapy with IFN-α and ribavirin^32^. If we cannot exclude a possible effect of treatments on PDE8A editing profile, it is likely that the decrease observed in PDE8A RNA editing reflects the changes due to the pathological status of patients. Activation of the immune system might also be involved in the differences observed here between DEP and SA, as illustrated by a trend in the increase of CRP in SA vs. DEP. Editing levels for sites B, C D, E, and F were strongly and positively correlated, suggesting that the modifications in editing levels occurs concomitantly within the whole editing island of PDE8A mRNA, the result being a global decrease in editing activity in patients’ blood. PDE8A isoform B, which is by far the major isoform observed in blood, was significantly decreased in patients, while at the same time an increase in the non edited isoform was observed, suggesting a modification in either ADAR expression, activity, or subcellular localization in depression. Moreover, the identified PDE8A RNA editing modifications strongly discriminated depressed suicide attempters form healthy controls.

In a previous work, PDE8A RNA editing profile measured in two brain regions showed that the non-edited isoform was quite stable between controls and suicide decedents^24^. In the blood of MDD patients, the increase in the non-edited isoform is in contrast with what we observed in the brain. In the brain, modifications in both the nature of isoforms and their relative abundance compensate each other, leading to no change in non edited isoform. In blood, we observed a global decrease in RNA editing activity in patients. In this case, the change in editing activity in PDE8A was not due to differential ADAR expression levels between groups, as measured by RT-qPCR. Previous studies suggested that ADARs mRNA and protein expression levels are not consistently associated with RNA editing levels. During brain development, editing levels of neurotransmitter receptors such as 5HTR2CR and GABAAR (*Gabra3)* increase, despite constant protein expression of ADAR proteins^38^. A recent report of A-to-I RNA editing signatures within the Drosophila brain identified editing differences between nine different neuronal populations, which were site-specific and not driven by changes in ADAR expression, suggesting a complex, targeted regulation of editing levels in key transcripts^39^. Indeed, spatial distribution of ADAR enzymes, and their interaction with partner proteins, which can act as inhibitor or activators, might influence ADARs’ editing activity^40^. As gene expression experiments did not show any modification in either PDE8A or ADARs editing enzymes, it is likely that the decrease might result from decreased ADARs activity and/or modified subcellular localization in patients.

A-to-I RNA editing is typically more frequent in brain than in other tissues, both in terms of number of editing sites and editing levels^41^. A recent survey of RNA editing in six different tissues confirmed that brain contained the highest number of tissue-specific RNA editing events^42^. Here, the PDE8A RNA editing levels for site B measured in blood were similar to those measured in brain, while editing levels for the four other sites considered were 5-fold less edited than in the brain. RNA editing is known to be strongly tissue-dependent; levels of RNA editing events might also be different in different cell types from the same tissue. It has been shown recently that the landscape of global RNA editing differs between brain and blood^43^. Characterization of RNA editing sites in human monocytes showed that RNA editing in blood cells was enriched in intronic region of repetitive elements, most of the sites being edited at lower frequency^43^. RNA editing might have crucial implications in non-brain tissues. As an example, a non-synonymous RNA editing event in the coding region of Filamin A pre-mRNA regulates vascular contraction and diastolic blood pressure and is associated with cardiovascular diseases^44^. Contrary to the recoding editing event in Filamin A, most edited sites are located in repetitive regions, and 90% occur in Alu elements^42,45^. They are usually found as adjacent inverted Alu elements, which form a stem-loop structure triggering ADARs binding^46^. It has been suggested that Alu elements function as editing inducer elements (EIEs), which recruit ADARs and contribute to efficient editing at specific sites^47^. The region studied here does not belong to an Alu element but rather to a non-repeated intronic element. As intron 9 of PDE8A also form a long stem-loop structure^24^, editing sites located in 3′ to site B, namely sites C, D, E, and F could also possibly serve as inducer elements for editing at site B.

Inflammation is known as a pathogenic factor, possibly explaining a part of physiopathology of depression^48^. Brain tissue from suicide decedents showed increased activation of microglia, which could release pro-inflammatory and anti-inflammatory mediators such as cytokines and chemokines upon activation (for review see ref. ^49^). The association between inflammation and depression also translates in blood. Common genetic variants, including polymorphisms in the genes IL-1β, IL-6, or CRP are involved in both immune activation and depression^50^. Many studies revealed that patients suffering from depression have increased circulating pro-inflammatory cytokines, such as TNF-α, IL-1β, or IL-6, suggesting that a neuroimmune axis interfacing the immune system and CNS might be involved in the control mood and behavior^51^. A genome-wide pharmacogenetic study in MDD patients identified single nucleotide polymorphisms in IL-11 and IL-6, which could predict antidepressant response^52^. A discovery approach to identify gene expression changes in suicidality identified a panel of 76 biomarkers, a subcategory of which have biological roles in immune and inflammatory response^53^. Recent data suggest that peripherally derived chemokines and cytokines can directly act in the brain, and that some immune cells can actually access the brain^54^. The chronic low-grade inflammation observed during depressive episodes can activate the hypothalamic-pituitary-adrenal (HPA) axis, resulting in an increase in cortisol levels^55^. Moreover, inflammation induced dysregulation of the tryptophan-kynurenine pathway, which contributes to the neurodegeneration observed in major depression and suicidality^56^.

Genome-wide analysis of RNA edited sites in human blood recently revealed a strong enrichment in genes involved in immune system and interferon signaling^40^. PDE8A is an immune response marker, RNA editing of which has been observed in brain and blood. RNA editing is altered in cancer, autoimmune, and neurological disorders (for review see ref. ^57^). A recent study reported an overall increased RNA editing in SLE^58^. In that study, patients could even be discriminated by the presence of the IFN-inducible genes’ expression signature. Interferons are crucial regulators of the immune system and are known to trigger the protective defenses of the immune system (for review see ref. ^59^). Importantly, Type I interferon can upregulate ADAR1-1 (ADAR1p150), and hence increase general RNA editing activity^37,60^. Treatment of a human glioblastoma cell line with IFN-alpha induced a 5-fold increase in ADAR1a, which was accompanied by significant changes in the editing of serotonin 2C receptor (5HT2CR) mRNA^61^.

Phosphodiesterases (PDEs) are the only enzymes hydrolyzing adenosine and guanosine 3′,5′-cyclic monophosphates (cAMP and cGMP, respectively), which are key modulators of signal transduction that mediate the cells’ response to a broad variety of hormones and neurotransmitters (for review see ref. ^25^). Different classes of antidepressants have been shown to increase cAMP pathway in both hippocampus and cortex^62^. PDE8A is expressed in different brain region, including BA9 and BA24, where it participates in serotonin signaling. PDE8A mRNA is twofold decreased in the temporal cortex of MDD patients^63^. Consistent with our data, Orlowski et al. showed decreased RNA editing in T lymphocytes from SLE patients and in T cells activated with IFN-alpha^27^, suggesting that increased ADAR expression following interferon response might not correlate with ADARs editing activity^40^. Interestingly, serotonin receptors are present on immune cells and serotonin has immune-regulatory effects (for review see ref. ^64^). Serotonin modulated the release of pro-inflammatory cytokines from monocytes, including IL-1β and IL-6, when stimulated by lipopolysaccharide^65^. Therefore, PDEs are very likely to play an important role in immune cells during inflammation. Noteworthy, PDE8A has been shown in vitro to physically interact with endogenous IκB proteins, and binding to IκB increased enzymatic activity of PDE8A^66^. Therefore, the transcription factor NF-κB, which is a pivotal mediator of inflammatory response might also participate directly to the regulation of second messenger levels, as cAMP-induced signals might interfere with NF-κB and modulate its actions^67^.

In conclusion, the data presented in this study identified PDE8A as a peripheral immune response-related marker previously documented in the brain. The observation of altered PDE8A RNA editing in the blood in depressed patients, combined with the identification of a combination of isoforms, that allowed discrimination between healthy controls and suicide attempters raises the possibility of providing a molecular signature, whose modifications in blood mirror those occurring in the brain. This suggests that both the central nervous system and the immune system act on the control of mood and behavior. A role of RNA editing in the non-coding intron of PDE8A in both brain and blood might suggest that this non coding intron might have functional role during or after splicing, yet to be identified. The finding that PDE8A RNA editing translated from brain to blood strengthen the association between inflammation and depression, and could pave the way for predictive blood-based biomarkers to evaluate depressive symptoms.

## Supplementary information

Supplementary_Material

Supplementary Figure 1

Supplementary Figure 2

Supplementary Figure 3

Supplementary Figure 4

Supplementary Figure 5
