# Supplementary material for: Phosphodiesterase 8A to discriminate in blood samples depressed patients and suicide attempters from healthy controls based on A-to-I RNA editing modifications
Source: Transl Psychiatry. 2021 Apr 30;11:255. doi: 10.1038/s41398-021-01377-9 (PMC8087806; doi:10.1038/s41398-021-01377-9)
Supplement: Supplementary file 1 — Supplementary_Material [file 41398_2021_1377_MOESM1_ESM.docx]

**Supplementary Figure legends**

Supplementary Figure 1: Ultra deep targeted sequencing workflow for measurement of RNA editing in exon 9 of PDE8A.

1. Total RNAs were extracted from PAXgene™ blood RNA tubes, qualified and quantified. PCR1 was run with specific primers containing Illumina adapters, and a second PCR product was generated with Illumina Nextera XT Index Kit. Purification using magnetic beads was performed after each step, as were Quality Controls, i.e. quantification and qualification of purity, for each PCR product. Library was pooled and denatured using 0.1N NaOH before loading on an Illumina NextSeq® 500/550 Mid Output Kit (300 cycles).
2. overview of bioinformatics workflow for Targeted Next Generation Sequencing

Supplementary Figure 2: Sequence of the 225bp amplicon in intron 9 of PDE8A mRNA analyzed in this study. Sequence of PCR primers are indicated in blue. Specific sites of RNA editing are highlighted in red.

Supplementary Figure 3: Correlation between clinical MADRS and IDS-C30 scores for all the patients included in the study. The Pearson correlation coefficient and p value are indicated on the graph.

Supplementary Figure 4: Principal Component Analysis on the first and second components, showing the administered groups of medications: (A) antidepressant, (B) antipsychotics, (C) anxiolytics, (D) hypnotics.

Supplementary Figure 5: Correlation between editing values in sites B, C, D, E and F of PDE8A mRNA in controls (A) and MDD (B) groups. Correlation plot visualize Pearson correlation matrix with the “corrplot R package”. Positive and significant correlation are in red square and correlation values are indicated.
