# Supplementary figures and images for: Phosphodiesterase 8A to discriminate in blood samples depressed patients and suicide attempters from healthy controls based on A-to-I RNA editing modifications
Source: Transl Psychiatry. 2021 Apr 30;11:255. doi: 10.1038/s41398-021-01377-9 (PMC8087806; doi:10.1038/s41398-021-01377-9)

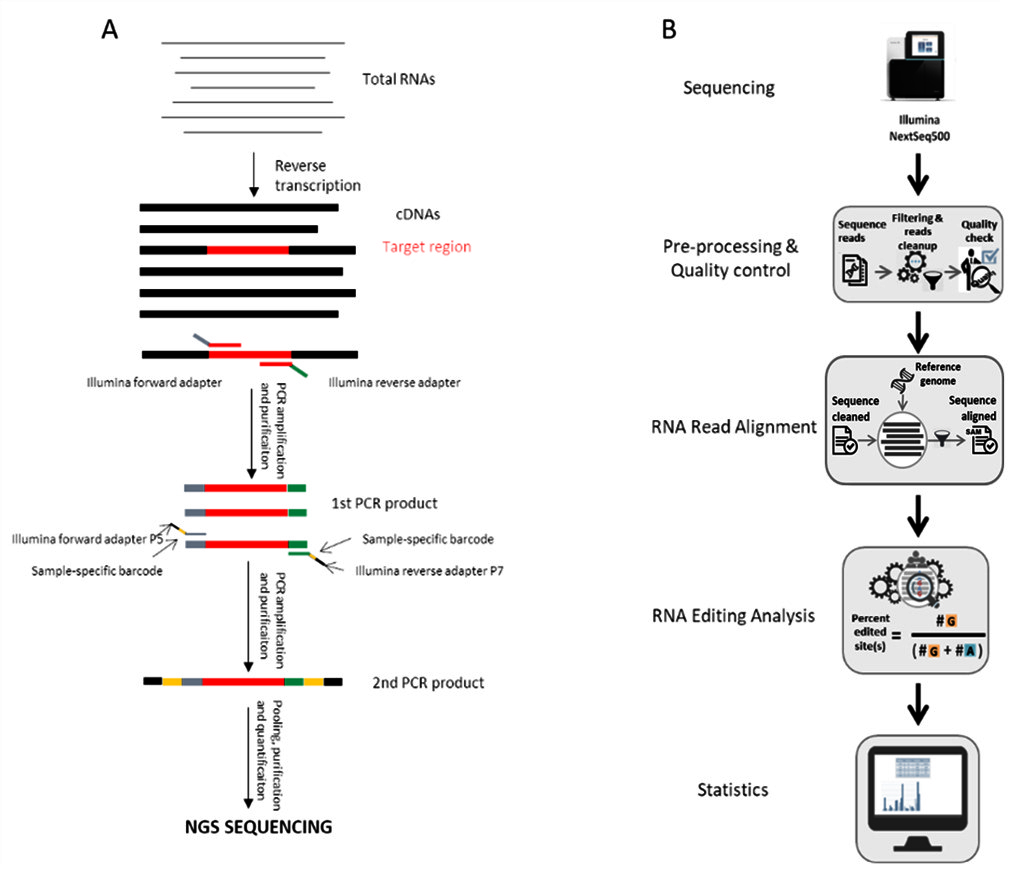

Supplement: Supplementary file 2 — Supplementary Figure 1 [file 41398_2021_1377_MOESM2_ESM.jpg]

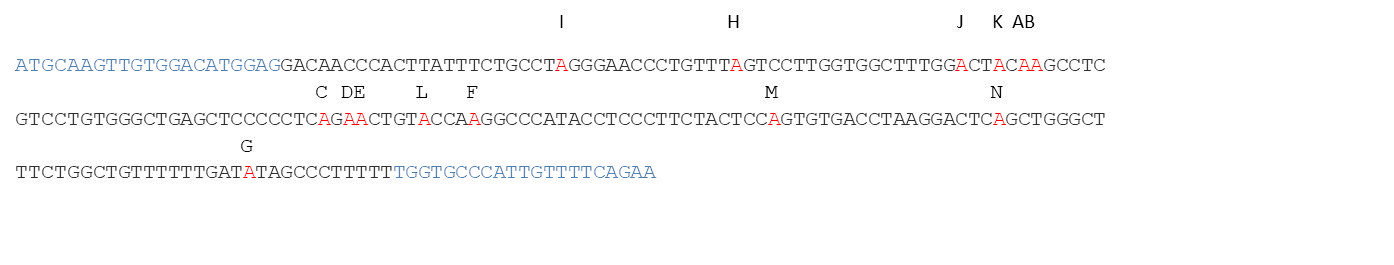

Supplement: Supplementary file 3 — Supplementary Figure 2 [file 41398_2021_1377_MOESM3_ESM.jpg]

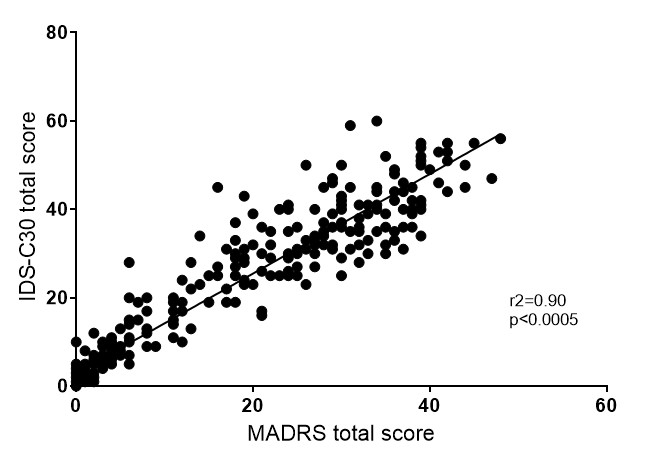

Supplement: Supplementary file 4 — Supplementary Figure 3 [file 41398_2021_1377_MOESM4_ESM.jpg]

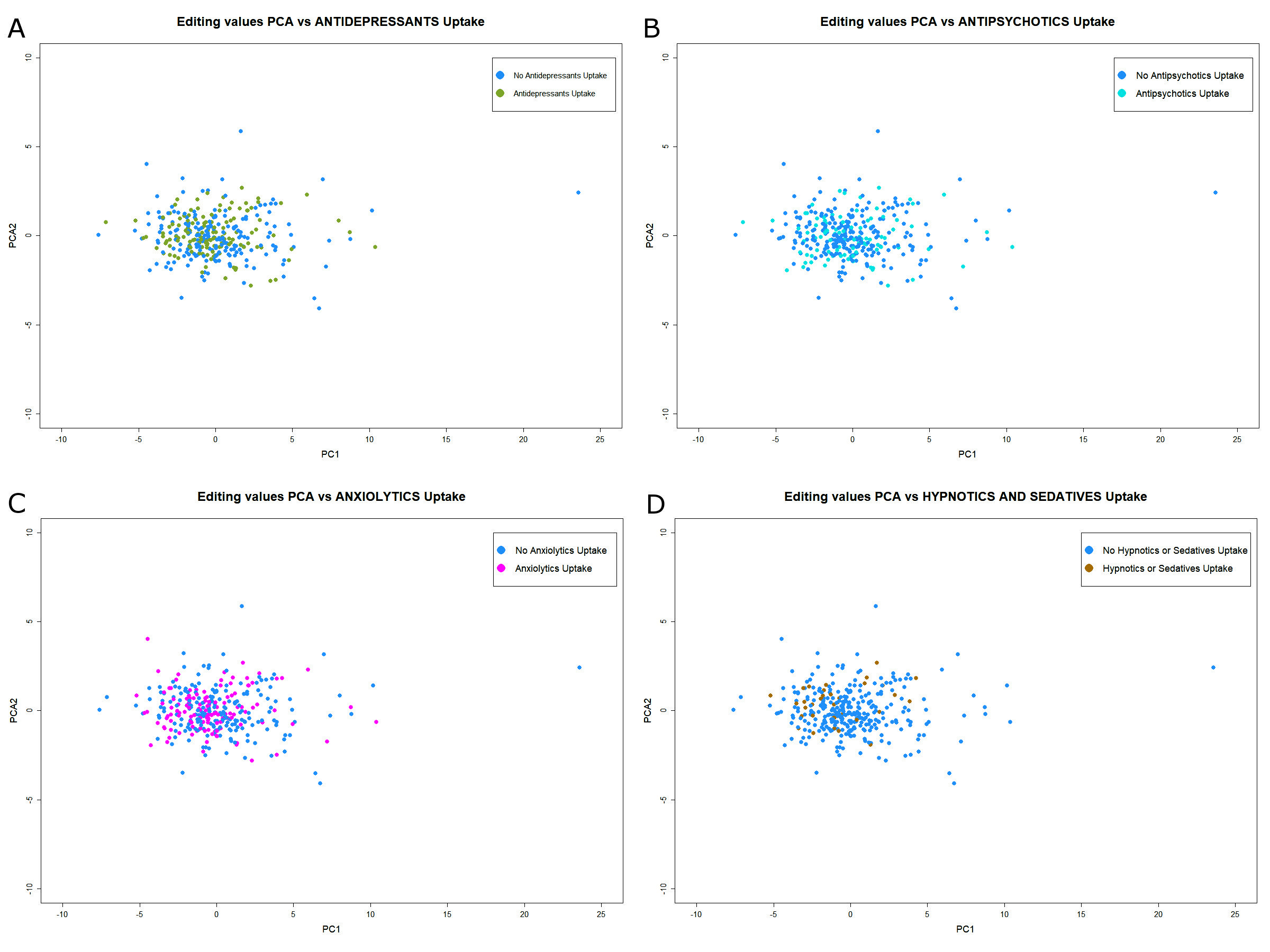

Supplement: Supplementary file 5 — Supplementary Figure 4 [file 41398_2021_1377_MOESM5_ESM.jpg]

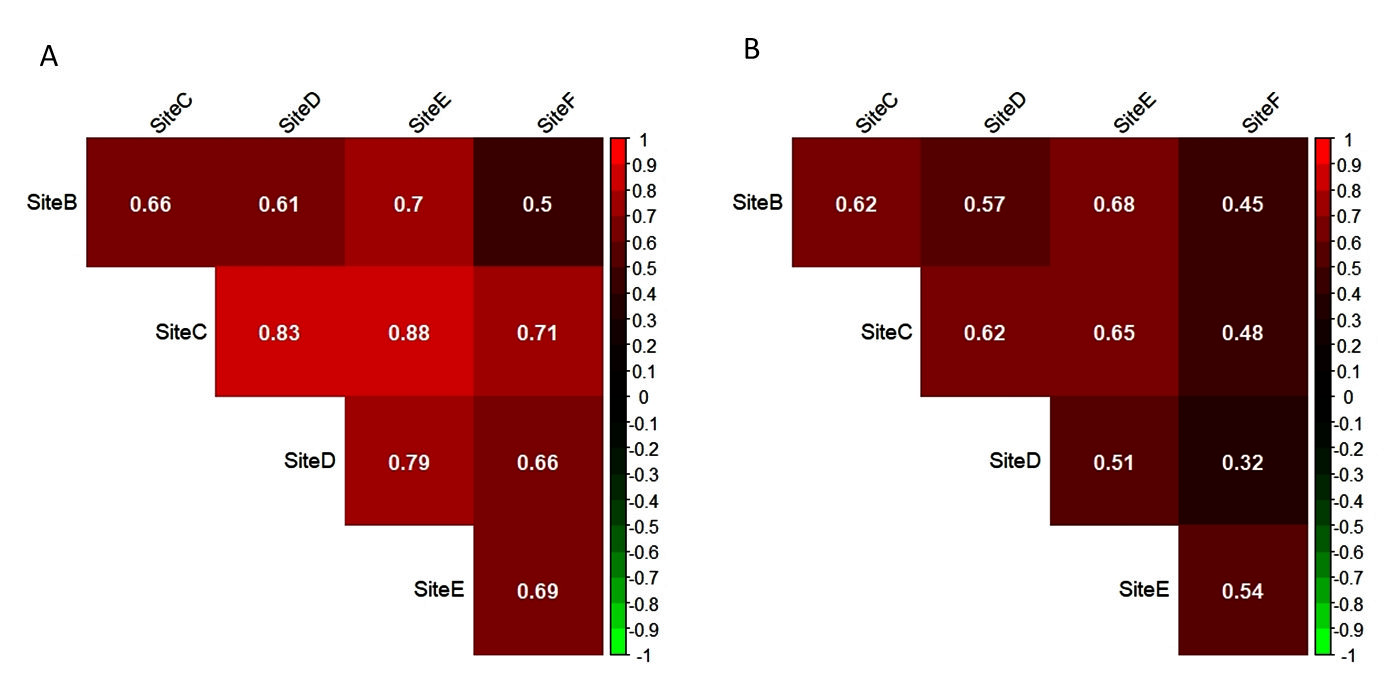

Supplement: Supplementary file 6 — Supplementary Figure 5 [file 41398_2021_1377_MOESM6_ESM.jpg]
